# Beyond transparency: why Traditional Chinese Medicine (TCM) need explainable artificial intelligence (XAI)

**DOI:** 10.1186/s13020-026-01455-4

**Published:** 2026-07-14

**Authors:** Wanting Zheng, Yuanyuan Tong, Jinjian Huang, Ling Zhu, Jiaqi Chai

**Affiliations:** 1 Institute of Information on Traditional Chinese Medicine, China Academy of Chinese Medical Sciences, Beijing, 100700 China; 2 College of Philosophy, Nankai University, Tianjin, 300350 China; 3 Institute of Basic Research in Clinical Medicine, China Academy of Chinese Medical Sciences, Beijing, 100700 China; 4 Institute of Science,Technology and Humanities, Shanghai University of Traditional Chinese Medicine, Shanghai, China

**Keywords:** Explainable artificial intelligence, Traditional Chinese Medicine, Epistemology, Syndrome differentiation, Semantic translation, Dual-layer opacity, Large language models, Knowledge graphs, Multimodal diagnosis

## Abstract

The integration of artificial intelligence (AI) with Traditional Chinese Medicine (TCM) is rapidly expanding, creating new opportunities for digital diagnosis, syndrome differentiation, prescription recommendation, multimodal clinical support, and knowledge mining. The central obstacle to this integration, however, lies beyond predictive performance and stems from a fundamental epistemological mismatch between data-driven AI and theory-driven TCM. Contemporary AI systems often operate through opaque statistical representations, whereas TCM depends on holistic, relational, and interpretive reasoning centered on syndrome differentiation and disease-mechanism inference. Consequently, explainable artificial intelligence (XAI) is required not merely to improve transparency but to serve as an epistemic interface that enables semantic translation between machine-discovered patterns and TCM clinical reasoning. This review argues that AI–TCM integration should be understood as an epistemological integration problem rather than a purely technical one. To address this problem, we introduce two conceptual lenses: the dual-layer opacity framework, which captures the superposition of algorithmic opacity and the theoretical opacity of TCM knowledge, and the semantic translation framework, which conceptualizes XAI as the process of mapping computational features and reasoning traces onto clinically meaningful and theory-consistent TCM concepts. On this basis, we critically synthesize major methodological pathways of TCM-XAI, including feature-attribution methods, visual explanation, intrinsically interpretable models, knowledge-guided reasoning, and large-language-model-based explanation infrastructures such as chain-of-thought and graph-based retrieval-augmented generation. Beyond methodological synthesis, we identify four core criteria for high-quality explanation in TCM: faithfulness, clinical relevance, theoretical coherence, and cultural integrity. We show that the principal challenges of TCM-XAI extend beyond accuracy and include annotation uncertainty, explanation validation, privacy-preserving interpretability, fairness across populations, and the risk of epistemic reduction or cultural misinterpretation. Finally, we outline a future research agenda centered on causal inference, neuro-symbolic reasoning, multimodal explanation benchmarks, clinician-centered evaluation, and regulatory standards for trustworthy TCM-AI systems. We argue that XAI should be understood not as a technical add-on but as the epistemic infrastructure required to build clinically trustworthy, culturally coherent, and scientifically robust AI ecosystems for Traditional Chinese Medicine.

**Supplementary Information:**

The online version contains supplementary material available at 10.1186/s13020-026-01455-4.

## Introduction

The integration of artificial intelligence (AI) with Traditional Chinese Medicine (TCM) represents a rapidly expanding frontier in contemporary medical research. AI is now applied to TCM diagnosis, syndrome differentiation, prescription recommendation, and other areas, while the emergence of domain-specific large language models has further stimulated interest in computational approaches to TCM modernization [1–6]. Surveys indicate that public and patient attitudes toward this integration are growing more favorable, though acceptance remains contingent on trust, interpretability, and respect for TCM's theoretical integrity [5].

Despite this momentum, integrating AI with TCM involves challenges beyond improving predictive performance. TCM is a theory-driven medical system grounded in holistic and interpretive reasoning, not merely a collection of empirical rules. As foundational accounts emphasize, its diagnosis is organized around patterns of disharmony rather than isolated disease entities, relying on a synthesis of observations within frameworks of qi dynamics, yin–yang balance, and zang-fu relationships [7]. Consequently, its diagnostic core, syndrome differentiation, constitutes a structured mode of clinical reasoning.

This distinction is crucial for AI applications. In contemporary machine learning, predictions often arise from high-dimensional statistical transformations whose internal logic remains largely inaccessible, a phenomenon described as algorithmic opacity or the black-box problem [8–11]. Predictive success alone does not ensure interpretability, trustworthiness, or meaningful human oversight. In healthcare, where decisions must be clinically actionable and ethically accountable, explanation is therefore a foundational requirement, not an optional enhancement [11–13].

Within TCM, the interpretability problem intensifies as machine opacity intersects with the distinctive epistemic structure of traditional medical reasoning. Clinicians require judgments interpretable in terms of syndrome evolution, disease mechanism, and treatment principle; a statistically convincing but theoretically unintelligible result holds limited value in a system whose authority rests on coherent links between observation and intervention. This challenge is compounded by the inherent inter-rater variability in TCM diagnosis, meaning training data may encode expert disagreement and contextual uncertainty [14, 15]. An AI system may consequently learn annotation habits or shortcut correlations rather than medically valid structure [16].

To capture this difficulty, we argue that AI–TCM integration should be understood through a dual-layer opacity framework (elaborated in section "The dual-layer opacity problem"). Briefly, the first layer is algorithmic opacity inherent in AI systems, and the second is theoretical opacity arising from the epistemological structure of TCM. Their overlap creates a compound barrier to validation, accountability, and clinical trust, explaining why concerns extend beyond technical reliability to cultural misinterpretation and the oversimplification of traditional reasoning [5].

These concerns have practical consequences. Research shows clinicians need explanations relevant to the decision context and aligned with domain reasoning, not merely post hoc visualizations or generic feature-importance scores [11, 13]. In regulatory settings, the importance of explanation is also growing, with AI systems increasingly evaluated for traceability, auditability, and accountability [12, 17]. Recent work on AI medical devices, including in China, indicates explainability is becoming more relevant to approval and responsible deployment [12].

Against this background, explainable artificial intelligence (XAI) should not be treated merely as a technical enhancement for transparency. In TCM, XAI must function as an epistemic interface: a structured means of translating machine-discovered patterns into clinically meaningful and theoretically coherent knowledge. The central challenge is therefore not simply to open the black box, but to establish a pathway of semantic translation between raw clinical data, computational feature space, and TCM concept space.

On this basis, this review makes three contributions. First, it proposes the dual-layer opacity framework (section "The dual-layer opacity problem"), which characterizes the superposition of algorithmic and theoretical opacity in AI–TCM integration. Second, it proposes a semantic translation perspective, reframing XAI as the process of mapping computational features onto clinically meaningful and theory-consistent TCM concepts. Third, it synthesizes current methodological pathways and application domains of TCM-XAI—from feature attribution to LLM-based explanation infrastructures—and outlines a future research agenda for building trustworthy, theory-aligned intelligent systems for TCM.

## Why TCM poses a special challenge for explainable AI

### TCM as a theory-driven and interpretive medical system

The need for explainability varies across medical fields, and it is particularly acute in Traditional Chinese Medicine (TCM), where diagnosis and treatment follow a pattern-based reasoning framework grounded in explicit theoretical constructs rather than isolated symptom-outcome correlations. Recent reviews document the rapid expansion of artificial intelligence into TCM diagnosis, syndrome differentiation, herbal prescription, knowledge mining, network pharmacology, multimodal fusion, and large-language-model-assisted reasoning [1–5, 18–21]. These same sources, however, reveal a persistent tension: while computational systems can process TCM data at scale, the clinical legitimacy of their outputs hinges on interpretability within TCM's conceptual logic, not merely the internal logic of statistical learning [1–5, 18–21].

This challenge stems from the epistemic structure of TCM. Foundational scholarship emphasizes that TCM operates not through reductionist disease labeling but through pattern-based reasoning, which interprets symptoms, signs, bodily states, environmental context, and temporal progression relationally [7]. In practice, the meaning of an individual sign is seldom fixed in isolation. A slippery pulse, a red tongue edge, a bitter taste, or emotional irritability gains diagnostic significance only when situated within a broader syndrome complex and interpreted in relation to pathogenesis, disease dynamics, and treatment principles. Consequently, TCM is not merely data-rich but theory-saturated.

Recent computational research reinforces this point. Large-scale TCM reviews from 2024–2026 indicate that despite increasingly sophisticated AI methods, their success remains limited by the difficulty of aligning low-level computational features with higher-level TCM constructs like syndrome, pathogenesis, and treatment logic [1–5, 18–21]. Investigations into multimodal fusion, network-based herbal reasoning, and domain-specific TCM language models all suggest that performance gains do not automatically yield gains in interpretability [4, 18–21]. This is precisely why TCM presents a distinct challenge for explainable AI: the core problem is not simply rendering predictions visible but ensuring they are meaningful within a theory-saturated clinical tradition.

### Why syndrome differentiation cannot be reduced to label prediction

This challenge is most evident in syndrome differentiation, the cognitive core of TCM practice. While often framed computationally as a classification problem, this approach risks flattening a complex inferential process into a simple label-assignment task. Clinically, the process involves moving from observed manifestations to an explanation of the disease mechanism, and from that explanation to a treatment principle and strategy. It therefore constitutes a form of structured reasoning rather than an ordinary prediction task. Recent benchmark development underscores this distinction. The TCM-SD benchmark provided an early public resource for probing syndrome differentiation in large-scale clinical data, while newer work such as TCMEval-SDT evaluates the reasoning process itself, not merely the final diagnostic output [22, 23]. Parallel efforts in prescription auditing and syndrome–treatment coupling further demonstrate a growing recognition that TCM-AI systems must be assessed for the coherence of their reasoning trajectory, not just answer correctness [24]. These developments substantiate a central claim of this review: modeling syndrome differentiation as a shallow pattern-recognition task risks epistemic distortion. A second complication is annotation instability. Both historical reliability studies and recent systematic analyses indicate that TCM diagnosis often exhibits limited inter-rater agreement, meaning different practitioners may assign different syndromes to the same case or justify similar conclusions using different evidence constellations [14, 15]. From a machine-learning perspective, this introduces label uncertainty and increases the risk that a model will learn annotation artifacts, institutional conventions, or shortcut features instead of medically valid structure [16]. Given that syndrome differentiation forms the interpretive bridge between symptoms and treatment, instability at this labeling stage can propagate errors throughout the entire AI pipeline.

### The dual-layer opacity problem

The integration of AI with TCM thus presents a distinctive challenge characterized by a dual-layer opacity. The first layer stems from the inherent opacity of the AI models themselves. Although deep neural networks, multimodal fusion architectures, graph-based systems, and large language models frequently deliver strong benchmark performance, they obscure the inferential pathways leading to their outputs [8–11, 13, 25]. This opacity already raises significant concerns in healthcare regarding trust, safety, accountability, and auditability [11–13, 17]; in TCM, these concerns are intensified because the model is tasked with engaging a knowledge system that is itself profoundly interpretive. The second layer arises from the opacity of TCM theory from the perspective of modern computational representation. Foundational TCM constructs—such as qi stagnation, deficiency–excess transformation, damp-heat, and the dynamic root–branch pathology relationship—do not map neatly onto ordinary, low-level measurable variables. Contemporary TCM-AI research consistently encounters this disconnect: whether in syndrome differentiation, pulse analysis, tongue diagnosis, or prescription recommendation, the computational features learned by models often remain difficult to reconcile with either biomedical variables or clinically accepted TCM concepts [1–5, 18–21, 26]. This does not imply TCM concepts are arbitrary, but rather that they belong to a different explanatory framework requiring interpretation rather than direct reduction. When these two forms of opacity intersect, they produce a unique epistemic difficulty. The core problem is not merely that the model is hard to interpret, but that the target medical reasoning is itself not directly representable in terms commensurate with the model’s internal features. This explains why explainability in TCM cannot be achieved through generic saliency maps, feature-importance rankings, or local surrogate approximations alone. While such techniques may reveal what a model attends to, they do not necessarily indicate whether its focus corresponds to a clinically meaningful syndromic process.

### Why XAI in TCM must support trust, translation, and theory refinement

Therefore, explainability in TCM must assume a broader role than is typically assigned in mainstream AI discourse. While discussions of XAI often emphasize transparency, trust calibration, or post hoc interpretability [10, 11, 27–29], these objectives, though necessary, are insufficient for TCM. XAI in this context must actively support three core functions: fostering clinical trust, enabling semantic translation, and facilitating theory refinement.

First, it must cultivate clinical trust. Research in medical XAI indicates that explanations can either enhance or diminish clinician trust, depending on their contextual appropriateness, faithfulness to the model, and relevance to the clinical workflow [11, 13]. Similar concerns emerge in TCM-focused surveys and reviews, where clinicians and patients alike worry about opaque reasoning, the oversimplification of traditional knowledge, and the unjustified overriding of practitioner judgment by algorithmic authority [5, 13]. Consequently, trust in TCM-AI systems cannot be established through performance metrics alone but must be rooted in reasoning that is inspectable and contestable.

Second, XAI must perform semantic translation. The model's computational features need to be rendered interpretable using clinical concepts familiar to practitioners, such as pulse quality, tongue morphology, disease tendency, treatment principle, or herb compatibility. Without this translational step, explanations remain trapped in machine-readable representations and cannot meaningfully inform human medical reasoning.

Third, XAI should enable theory refinement. A promising argument in recent TCM-AI literature is that AI can help externalize latent diagnostic structures, uncover hidden symptom–herb associations, and identify previously unrecognized regularities in clinical data [2–5, 19–21, 30]. These discoveries only become scientifically productive, however, when the identified patterns can be interpreted, debated, and validated. Thus, XAI serves not merely as a risk mitigation tool but also as a disciplined mechanism for knowledge generation.

**Fig. 1:**
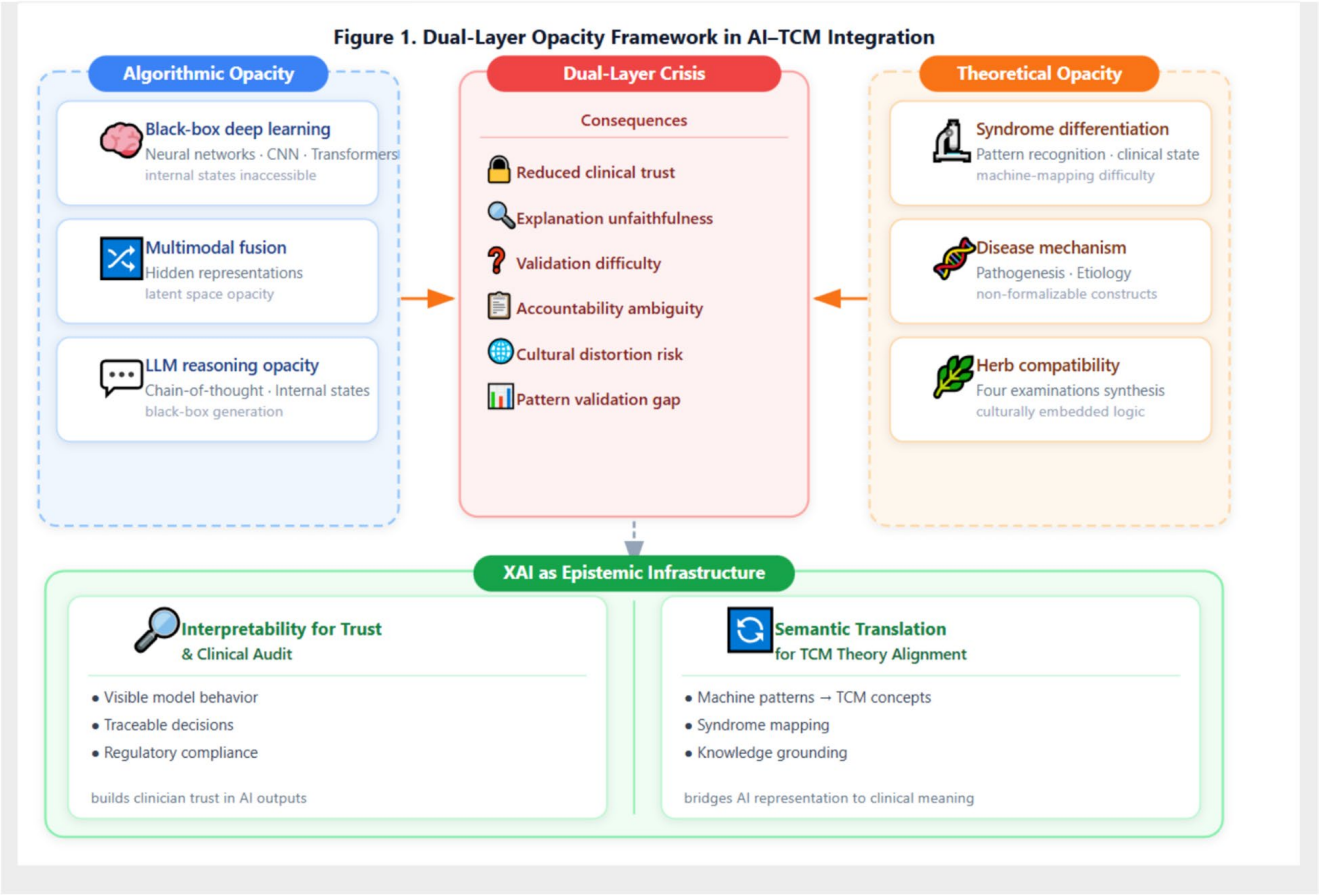
Dual-layer opacity framework in the integration of artificial intelligence and Traditional Chinese Medicine

Accordingly, this review argues that TCM requires not just more accurate AI, but AI whose outputs can be translated, validated, contested, and integrated within its theory-driven tradition. This is why XAI must be viewed not as a secondary technical add-on, but as a translational bridge between computational reasoning and clinical practice, serving as the essential methodological prerequisite for meaningful AI–TCM integration.The dual-layer opacity framework and its implications for trustworthy AI–TCM integration are summarized in Fig. 1.

The core challenge of this integration is conceptualized here as a superposition of two forms of opacity. Algorithmic opacity refers to the inherent inaccessibility of contemporary AI systems like deep neural networks and large language models. Theoretical opacity denotes the difficulty of directly mapping TCM constructs—such as syndrome, disease mechanism, and treatment principle—onto machine-readable variables or modern biomedical representations. The interaction of these opacities creates a dual-layer crisis, manifesting as reduced clinical trust, limited explanation faithfulness, difficulty in validating model-discovered patterns, ambiguous accountability, and the risk of cultural or conceptual distortion. This framework clarifies why explainable AI in TCM cannot be treated merely as a transparency accessory, but must function as the epistemic infrastructure necessary for trustworthy integration.

## From explainability to epistemic translation

### The limits of generic XAI in TCM

Mainstream explainable artificial intelligence methods were developed primarily to address general interpretability challenges in machine learning, not the specific epistemic demands of Traditional Chinese Medicine. Techniques like SHAP, LIME, saliency mapping, attention visualization, and local surrogate modeling can identify predictive variables, reveal an image model's focus, or illustrate a classifier's behavior under perturbation [27–29, 31]. While these tools remain valuable and widely used in medical AI, they often constitute only a preliminary step in TCM applications because they emphasize model transparency over medical intelligibility. As the dual-layer opacity framework (section "The dual-layer opacity problem") clarifies, a model might indicate statistically important features yet still fail to explain how those features correspond to syndrome evolution, disease mechanisms, or treatment principles in clinically meaningful terms [10, 18–21, 27–29].

Recent reviews of TCM-AI increasingly highlight this limitation. Large-scale syntheses published from 2024 to 2026 consistently identify the primary bottleneck not as prediction accuracy, but as the gap between computationally extracted patterns and TCM's conceptual vocabulary [1–5, 18–21]. This gap is evident across diagnostic modalities. In tongue diagnosis, a heatmap might highlight a discriminative region without clarifying whether it corresponds to a clinically meaningful tongue sign or merely a visual artifact. For pulse diagnosis, a model may assign high importance to specific waveform segments or spectral coefficients without explaining how these map onto qualitative pulse descriptors like wiry, slippery, or thready. In syndrome differentiation, feature-attribution methods can identify symptom combinations associated with a syndrome label while leaving it unclear whether the model has captured the underlying pathomechanistic logic [22–24, 26, 32–36]. These examples demonstrate that generic XAI, while successful at elucidating model behavior, does not yet ensure epistemic integration with TCM reasoning.

### A semantic translation framework for TCM-XAI

To address this gap, this review proposes a semantic translation framework for TCM-XAI, which defines explainability not as post hoc visibility but as the process of linking three representational spaces: the data space, the computational feature space, and the TCM concept space. The data space comprises heterogeneous, multimodal inputs, including tongue images, pulse waveforms, textual symptom descriptions, structured case records, prescriptions, laboratory indicators, and longitudinal clinical data [18–21, 37–43]. In the computational feature space, these data are transformed into latent embeddings, attention distributions, graph paths, token-level reasoning traces, frequency-domain descriptors, or multimodal fusion representations, which constitute the operational domain of modern AI models. By contrast, the TCM concept space is the domain where clinicians reason using syndromes, pathogenesis, treatment principles, herb compatibility, constitutional patterns, and disease dynamics. Consequently, the central challenge for XAI in TCM is not merely explaining the model's computations but mapping those computations into concepts that clinicians and researchers can interpret within a coherent TCM framework. This translation problem is increasingly evident in recent system designs. For instance, OpenTCM integrates a TCM knowledge graph with GraphRAG to ground large language model outputs in a structured semantic substrate, moving beyond reliance on free-form generation alone [44]. Its design rationale highlights that the core issue extends beyond hallucination control to ensuring that diagnostic question answering and knowledge retrieval remain anchored in interpretable concept relations derived from classical and domain-specific TCM knowledge. A similar focus is apparent in benchmark construction, as illustrated by TCMEval-SDT, which was explicitly designed to evaluate the thought process underlying syndrome differentiation and thus shifts emphasis from endpoint correctness to reasoning structure [23]. In TCM, an explanation holds value only if it can effectively mediate between computational reasoning and the clinician's interpretive logic.

### Translation across modalities: image, waveform, text, and graph

The semantic translation framework clarifies why distinct tasks in TCM demand different explanatory forms. For image-based diagnosis, such as tongue or face analysis, the problem is primarily spatial-semantic: models detect regions, textures, colors, or morphological features, but these outputs gain clinical meaning only when linked to recognized tongue or facial signs and subsequently to syndrome interpretation [37–39, 45–47]. In pulse diagnosis, the translation is tactile-to-computational; while TCM pulse assessment stems from embodied sensory experience, AI models typically represent pulse data through waveform morphology, time‑frequency decomposition, or learned latent features. Recent machine‑learning studies that integrate pulse diagnosis with modern signal analysis demonstrate progress, yet they also highlight the persistent difficulty of mapping computational descriptors back to clinically credible pulse categories and, further, to pathomechanistic interpretation [40, 41].

For text‑based syndrome differentiation and clinical reasoning, the problem becomes inferential‑semantic: large language models and benchmarks like TCMEval‑SDT increasingly focus on whether systems can articulate intermediate reasoning steps. However, recent TCM LLM studies reveal that models can generate linguistically plausible yet epistemically weak reasoning chains if those chains lack grounding in knowledge structures, domain constraints, or validated syndrome logic [23, 24, 26, 32, 33, 48–55]. Thus, the central explanatory question is whether a system’s reasoning trace is clinically faithful and theory‑consistent, not merely articulate. In knowledge‑graph and prescription systems, the translation problem is relational‑semantic; path‑based reasoning, herb‑symptom graphs, and graph‑guided retrieval enhance readability by externalizing relations that practitioners already find meaningful [26, 56–63]. Here, explanation entails making the underlying relational structure inspectable and open to critique.

### What counts as a good explanation in TCM?

**Fig. 2:**
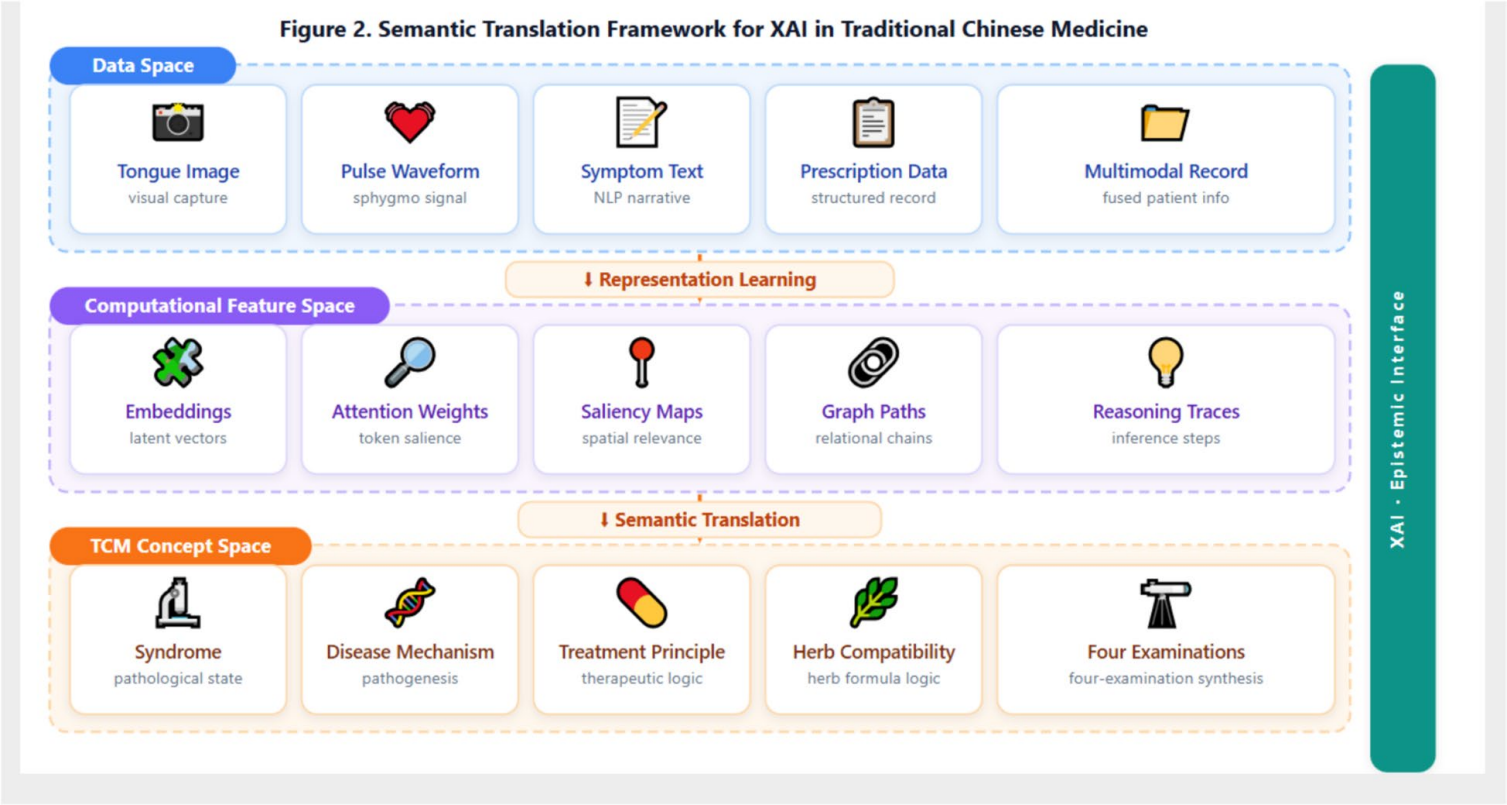
Semantic translation framework for explainable artificial intelligence in Traditional Chinese Medicine

If explainability in TCM is framed as semantic translation, its quality cannot be assessed by visibility alone. A high-quality explanation must satisfy four criteria: faithfulness, clinical relevance, theoretical coherence, and cultural integrity. First, it must be faithful, genuinely reflecting the model's operative behavior rather than offering a persuasive but disconnected narrative [10, 13, 27–29]. Second, it must be clinically relevant, meaning it addresses practitioners' real informational needs by indicating whether the model's intermediate evidence corresponds to recognizable signs and whether its syndrome logic is inspectable [11, 13]. Third, the explanation must be theoretically coherent, interpretable within TCM's conceptual logic instead of remaining a purely mathematical account of feature influence [1–5, 7, 18–21, 26, 32, 33]. Fourth, it must preserve cultural and epistemic integrity; TCM is not merely a clinical system but a historically embedded knowledge tradition. As TCM-AI systems increasingly employ LLMs, GraphRAG, and knowledge-grounded retrieval, the critical question becomes whether they can retrieve or generate TCM knowledge without epistemic distortion [5, 48–55, 64–71]. Consequently, XAI in TCM is best understood not as a transparency accessory but as an indispensable translational interface. Its purpose is to render machine reasoning inspectable, translatable, and contestable within a traditional medical system whose diagnostic authority relies on the coherence between observation, theory, and treatment. The proposed semantic translation framework linking data, computational features, and TCM concepts is illustrated in Fig. 2.

This figure frames explainability in TCM as a process of semantic translation across three representational spaces. The data space comprises heterogeneous inputs, such as tongue images, pulse waveforms, symptom narratives, structured case records, prescription data, and multimodal patient information. Within the computational feature space, AI models transform these inputs into latent representations, including embeddings, attention weights, saliency maps, graph paths, and reasoning traces. The TCM concept space, in turn, contains the clinical interpretations of illness, which are articulated through syndromes, disease mechanisms, treatment principles, herb compatibility, and the logic of the four examinations. XAI functions as an epistemic interface that connects computational representations to clinically meaningful and theory-consistent TCM interpretations. Consequently, the figure underscores that a useful explanation in TCM constitutes not merely visible model behavior, but a successful translation of machine-derived patterns into interpretable medical reasoning (Table 1).

**Table 1:** Core conceptual distinctions in TCM-oriented explainable artificial intelligence

Concept	Definition in this review	Why it matters in TCM	Representative challenge
Algorithmic opacity	Limited transparency of internal AI decision pathways	Weakens trust in deep learning, multimodal systems, and LLM-based outputs	Black-box syndrome prediction
Theoretical opacity	Difficulty of mapping TCM concepts onto machine-readable or biomedical variables	Makes validation of AI-discovered patterns difficult	Disease mechanism interpretation
Dual-layer opacity	Superposition of algorithmic opacity and theoretical opacity	Defines the core epistemological crisis in AI–TCM integration	Trust and accountability gap
Semantic translation	Mapping from computational representations to clinically meaningful TCM concepts	Enables explanations that practitioners can understand and verify	From saliency map to tongue sign
Clinically faithful reasoning	Reasoning that is inspectable, evidence-linked, and clinically valid	Needed for syndrome differentiation and treatment planning	LLM-generated but weakly grounded logic
Theoretical coherence	Consistency of explanation with TCM conceptual logic	Prevents reduction of TCM to mere statistical shortcuts	Syndrome-to-treatment mismatch
Cultural integrity	Preservation of TCM conceptual and historical identity during AI integration	Avoids epistemic distortion and cultural misinterpretation	Misreading metaphorical TCM concepts

This table summarizes the core concepts introduced in this review and clarifies their definitions, relevance to TCM, and representative challenges in AI–TCM integration. It serves as a conceptual scaffold for the epistemological argument of the review.

## Methodological pathways of XAI in TCM

### From method listing to methodological taxonomy

Explainable artificial intelligence in Traditional Chinese Medicine should not be approached as a mere collection of technical tools. Given the substantial differences in modality, reasoning structure, and theoretical semantics across TCM tasks, XAI methods must be assessed based on the type of explanation they generate, the specific clinical task they support, and their capacity to translate computational features into TCM concepts [10, 18–21, 27–29]. Recent reviews of AI in TCM suggest a shift from prediction-centric models toward explanation-aware, multimodal, and knowledge-guided systems, yet methodological discussions remain fragmented and overly focused on tools [1–5, 18–21]. Consequently, this review categorizes TCM-XAI into four principal pathways: feature-attribution explanations, visual and spatial explanations, intrinsically interpretable or process-structured models, and knowledge-guided relational explanations [27–29].

This taxonomic framework offers advantages over a simple listing of methods for two key reasons. First, a single XAI tool can fulfill distinct functions in different TCM tasks. For instance, SHAP may explain tabular syndrome predictions, multimodal clinical outcome models, or extracted pulse-wave features, yet the semantic adequacy of these explanations varies considerably by context [28, 29, 35, 36]. Second, different TCM tasks require different forms of explanation. A saliency map might prove useful for tongue diagnosis but inadequate for prescription recommendation; a graph path could aid herb recommendation but be less informative for interpreting raw pulse waveforms [45–47, 56–63]. Therefore, the central question is not whether a method is generically explainable, but whether it is aligned with the task, clinically relevant, and consistent with TCM theory in specific applications [10, 11, 27–29].

### Feature-attribution explanations: LIME, SHAP, and structured clinical reasoning

Among current XAI methods, LIME and SHAP are the predominant model-agnostic approaches in TCM applications, as many critical tasks involve structured records, symptom tables, syndrome labels, or multimodal feature sets rather than end-to-end image prediction alone [27–29]. LIME explains individual predictions by fitting a local surrogate model to the instance, whereas SHAP quantifies feature contributions using a Shapley-value framework with a stronger mathematical foundation [28, 29]. In TCM research, these methods are valuable because they can reveal which symptoms, signs, laboratory indicators, syndrome scores, or demographic variables most strongly influence a diagnostic or therapeutic prediction. A recent study on TCM efficacy prediction in Long COVID exemplifies this application. By integrating machine learning with SHAP and nomogram analysis, the work identified clinically interpretable features—including chest tightness, aversion to cold, syndrome score, and inflammatory markers—as major contributors to treatment-response prediction, thereby clarifying the model's logic for clinicians [35]. Similarly, an explainable model for predicting heat syndrome in acute ischemic stroke has delineated the clinical variables driving syndrome classification, providing an initial step toward interpretable syndrome-oriented modeling [36]. Machine learning models that differentiate cold and hot syndrome in viral pneumonia further demonstrate how structured feature attribution can reveal clinically meaningful combinations of variables rather than isolated factors [34]. These methods nevertheless have important limitations. Feature-attribution techniques explain a model only after its feature space has been engineered or learned. If the original representation distorts TCM reasoning, the explanation may be mathematically sound yet clinically superficial [27–29]. Furthermore, feature importance does not equate to relevance for disease mechanisms. Consequently, while feature-attribution methods are useful for interpreting structured models, they are insufficient for fully translating model logic into theory-driven clinical reasoning.

### Visual and spatial explanations: Grad-CAM and its variants in tongue and facial diagnosis

For image-based TCM tasks, particularly tongue and facial diagnosis, visual saliency methods represent the predominant explainable AI approach. The highly influential Grad-CAM technique establishes a key baseline by producing class-specific localization maps through gradient propagation to convolutional feature maps. In TCM imaging applications, Grad-CAM is valuable for offering an intuitive means to assess whether a model attends to clinically relevant regions, including tongue edges, coating areas, fissures, or local discoloration [37–39, 45–47]. Subsequent research reinforces this utility. Quantitative interpretability analyses of CNN-based medical image models underscore that saliency methods can help separate clinically meaningful attention from spurious background bias [45]. Models developed for coated tongue recognition illustrate how heatmap explanations can reveal a focus on tongue surface patterns over irrelevant image artifacts [46]. More directly pertinent to TCM multimodal diagnosis, an explainable dual-modal model utilizing tongue and facial features demonstrated that gated multimodal fusion, coupled with interpretable feature analysis, can identify the specific visual cues driving the final prediction [47]. Nevertheless, standard saliency methods possess recognized limitations. Their localizations are often coarse, diffuse, and insufficiently sensitive to the subtle image details critical for TCM tongue diagnosis. Clinically significant distinctions frequently rely on fine-grained features like peeled coating, red dots, fissures, local edge color, or distended sublingual veins [37–39, 45–47]. Visual explanation alone is thus inadequate. While a saliency map can indicate where a model focuses, it cannot inherently convey the TCM-specific significance of that region.

### Intrinsically interpretable and process-structured models

An important alternative to post hoc explanation is to design models whose internal structure preserves an interpretable reasoning process. This approach is particularly well-suited to TCM, as many clinical tasks follow an explicit sequence: collecting symptoms, inferring a syndrome, selecting a treatment principle, and finally generating a prescription. A model that maintains this sequence can provide more meaningful explanations than a black-box predictor augmented with local attribution methods.

A strong example is PresRecST, which explicitly integrates syndrome differentiation and treatment planning into its prescription recommendation pipeline [56]. Instead of mapping symptoms directly to herbs, the model introduces intermediate states that correspond to these clinically meaningful reasoning steps. This design enhances the cognitive relevance of the output for practitioners, who can inspect the plausibility of the intermediate syndrome judgment and therapeutic logic before considering the final formula [56].

Recent benchmarking of TCM-specific LLMs reinforces this perspective. Benchmarks like TCM-SD and TCMEval-SDT increasingly assess not only output correctness but also the quality of the syndrome differentiation reasoning itself [22, 23]. This implies that future TCM-AI systems will be evaluated on their preservation of inferential structure, not merely their ability to produce plausible answers. Consequently, the pursuit of intrinsic interpretability in TCM should extend beyond traditional glass-box models like decision trees to include process-structured architectures that maintain clinically meaningful intermediate states.

### Knowledge-guided and relational explanations: knowledge graphs, GNNs, and graph-based reasoning

Among all XAI pathways, knowledge-guided and graph-based explanations align most naturally with TCM. TCM reasoning is inherently relational, connecting symptoms to syndromes, syndromes to pathogenesis, pathogenesis to treatment principles, and these principles to herb compatibility and formula construction. This relational nature makes knowledge graphs and graph neural networks particularly attractive, as they explicitly model relational structures rather than keeping them latent. Recent TCM research strongly supports this direction. For tinnitus diagnosis, a neighbor-augmented knowledge graph combined with explainable AI provided transparent syndrome inference by explicitly revealing patient similarity and graph-based reasoning paths [57]. In herb recommendation, meta-path guided graph attention networks have connected symptoms, syndromes, and herbs through interpretable paths, yielding explanations that more closely resemble clinical reasoning than isolated feature-importance scores [58]. Models employing multi-layer information fusion, multi-graph convolution, and semantic knowledge fusion for herb recommendation further underscore the critical role of explicit graph structure in interpretable therapeutic reasoning [59–63]. Knowledge-guided systems are especially vital for TCM because they bridge the gap between computational architecture and domain knowledge. Unlike purely latent models, they can reveal why a herb is recommended, how a syndrome links to symptoms, or how a treatment principle derives from a pathogenesis pattern. Their effectiveness, however, depends heavily on meticulous ontology design, relation curation, and concept normalization [26, 57–63]. Nevertheless, among current XAI approaches, knowledge-guided explanation remains one of the most promising pathways for enabling genuine semantic translation in TCM.

### Method–task alignment matters more than tool popularity

**Fig. 3:**
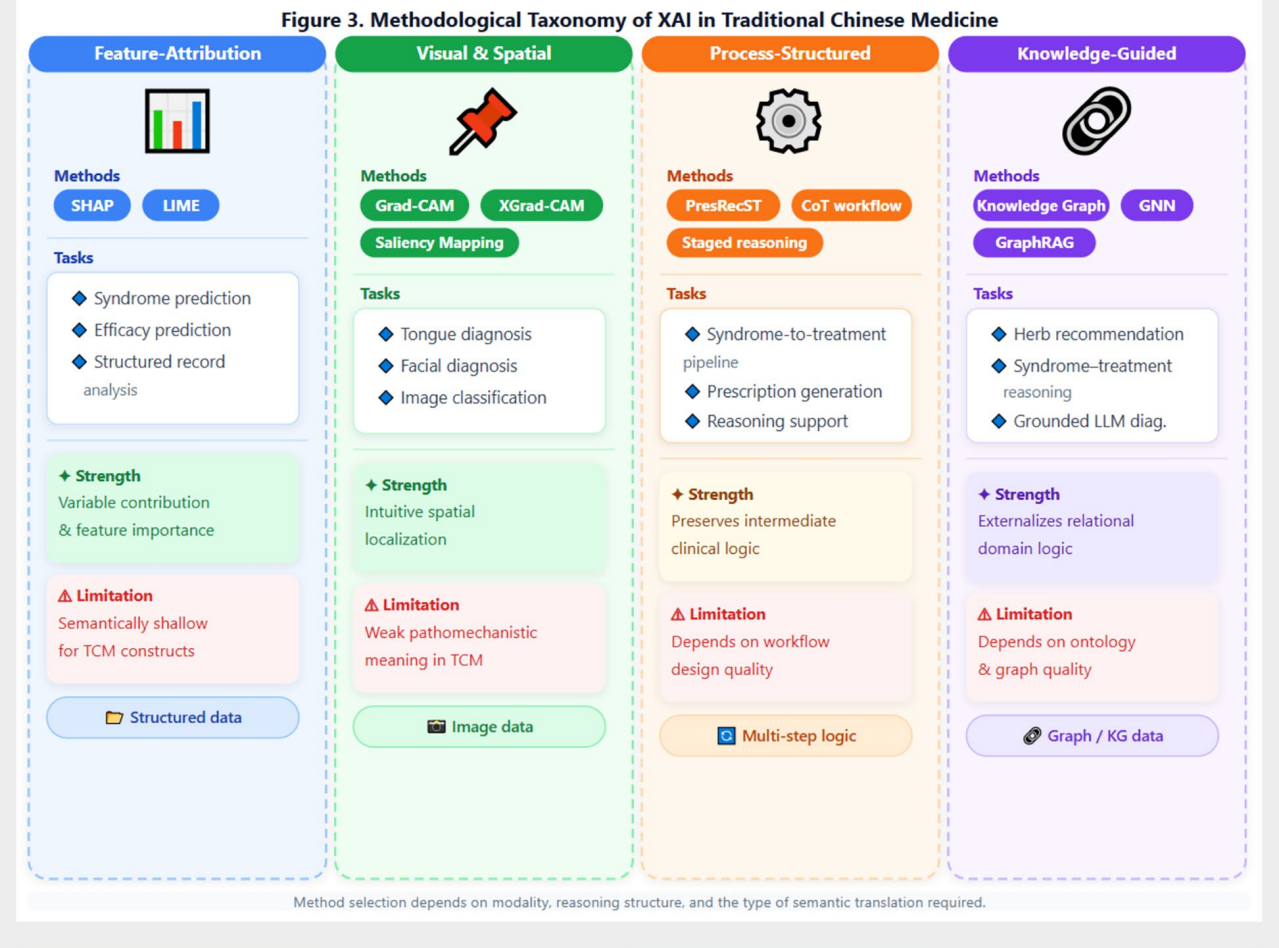
Methodological taxonomy of explainable artificial intelligence in Traditional Chinese Medicine

The literature indicates that no single XAI method is universally optimal for TCM; instead, the critical factor is the alignment between the explanation method and the specific clinical or epistemic task. For structured clinical prediction, efficacy modeling, and syndrome scoring, feature-attribution methods like SHAP are often most useful [34–36]. Visual explanation methods remain the most appropriate for tongue and facial diagnosis [37–39, 45–47]. In syndrome–treatment–prescription pipelines, process-structured models such as PresRecST provide higher cognitive relevance by preserving clinically interpretable intermediate reasoning [56]. For diagnosis and recommendation tasks grounded in domain knowledge, knowledge-guided relational explanations offer the best match, as they expose graph paths, concept relations, and explicit reasoning chains [26, 57–63]. This task-sensitive perspective explains why many disappointing explanations in TCM stem not from algorithmic failures, but from a mismatch between method and task. Consequently, the future of TCM-XAI depends less on identifying a universally superior method than on constructing explanation pipelines explicitly tailored to the representational, clinical, and epistemic demands of specific TCM tasks. The task-aligned taxonomy of TCM-XAI methods is summarized in Fig. 3.

Current TCM-XAI approaches can be categorized into four principal pathways. Feature-attribution methods quantify the contribution of structured variables and are frequently applied in syndrome prediction and efficacy modeling. Visual and spatial techniques identify discriminative regions within medical images, most notably for tongue and facial diagnosis. Process-structured or intrinsically interpretable models maintain clinically meaningful intermediate states, like syndrome differentiation and treatment planning, which proves valuable in staged reasoning pipelines and prescription generation. Knowledge-guided relational approaches externalize domain structure using knowledge graphs, graph neural networks, and graph-based retrieval, rendering them particularly apt for herb recommendation, syndrome–herb reasoning, and grounded LLM systems. The figure illustrates that no single XAI method is universally optimal for TCM; selection must instead be guided by data modality, reasoning structure, and the required form of semantic translation (Table 2).

**Table 2:** Comparative taxonomy of XAI methods in Traditional Chinese Medicine

XAI category	Representative methods	Primary data modality	Typical TCM tasks	Main explanatory strength	Main limitation	Suitability for semantic translation
Feature-attribution explanation	SHAP, LIME	Tabular, structured clinical data, mixed features	Syndrome scoring, efficacy prediction, structured case analysis	Reveals variable contribution and symptom importance	Often semantically shallow; dependent on pre-defined feature space	Moderate
Visual and spatial explanation	Grad-CAM, XGrad-CAM, saliency maps	Images	Tongue diagnosis, facial diagnosis	Intuitive localization of model attention	Limited pathomechanistic meaning; coarse localization	Moderate
Process-structured explanation	PresRecST, staged reasoning pipelines, intermediate-state modeling	Mixed clinical pipelines	Syndrome-to-treatment reasoning, prescription generation, LLM reasoning support	Preserves clinically meaningful intermediate steps	Requires carefully designed workflow and domain-aligned decomposition	High
Knowledge-guided relational explanation	Knowledge graph, graph neural network, GraphRAG	Graph, text, multimodal records	Herb recommendation, grounded diagnosis, syndrome–herb reasoning	Externalizes domain relations and evidence chains	Strong dependence on ontology completeness and graph quality	High

This table compares the principal categories of XAI methods used in TCM according to explanatory mechanism, supported data modality, representative clinical tasks, major strengths, major limitations, and suitability for semantic translation into TCM concepts.

## Large language models in TCM: from knowledge recall to clinically faithful reasoning

### LLMs have changed the form of explainability, not removed the need for it

The rapid emergence of large language models has significantly altered the landscape of explainable AI in traditional Chinese medicine. Earlier TCM-AI systems were primarily evaluated through classification accuracy, visual saliency, or feature-attribution analysis. In contrast, LLM-based systems can generate syndrome explanations, conduct multi-turn inquiries, summarize treatment logic, and recommend prescriptions in natural language, creating an impression of inherent interpretability [4, 19, 26, 32, 33, 48–55]. However, recent reviews of LLMs in TCM and integrative medicine consistently caution that linguistic fluency should not be conflated with clinically sound reasoning; a model may appear interpretable while remaining poorly grounded, theoretically inconsistent, or unsafe for clinical use [4, 26, 32, 33, 48–55]. This shift indicates that the explainability problem has not been resolved but has instead transformed. For earlier deep-learning systems, the central challenge was to render hidden feature transformations visible. In LLM-based systems, the critical question is whether a generated explanation reflects genuine domain reasoning or merely persuasive text generation [23, 24, 26, 32, 33, 48–55]. This distinction is particularly vital for TCM, where diagnostic legitimacy relies heavily on interpretable chains of reasoning.

### The key empirical problem: knowledge recall is not the same as clinical reasoning

Recent benchmark studies have quantified this gap. In an evaluation of LLMs on stroke-related TCM tasks, DeepSeek-R1 surpassed GPT-4o on objective questions demanding precise recall of TCM facts, whereas GPT-4o excelled at subjective tasks requiring integrated clinical reasoning and classical-text interpretation [32]. This finding is critical for TCM-XAI, as it demonstrates that accurate recall of herb properties, flavors, meridian tropism, or formula facts does not ensure robust syndrome-differentiation reasoning. A similar pattern emerges across recent TCM-domain models. Qibo introduced both a domain-adapted TCM model and a benchmark that explicitly separates subjective, objective, and TCM-specific NLP tasks, revealing that benchmark structure itself influences assessments of a model's capabilities [54]. BianCang employed a two-stage inject-then-align training paradigm to enhance syndrome differentiation and diagnostic performance [55]. Tianyi developed a TCM all-rounder model alongside a TCM-specific evaluation framework explicitly targeting exams, clinical tasks, and domain QA [33]. These developments confirm that domain adaptation improves TCM performance, yet it alone cannot guarantee clinically faithful reasoning.

Since this early benchmark, the LLM landscape for TCM has expanded considerably. The TCM-5CEval benchmark evaluated 15 LLMs across five dimensions—core knowledge, classical literacy, clinical decision-making, Chinese materia medica, and non-pharmacological therapy—and confirmed DeepSeek-R1 among the top performers, alongside Gemini 2.5 Pro and Kimi_K2 [72]. TCM-5CEval also revealed that all models exhibit positional sensitivity: permutation consistency scores dropped from 0.787 to 0.470 when answer order was changed, indicating that even top-performing models rely partly on surface-level pattern matching rather than robust understanding [72]. A subsequent five-model comparison assessing TCM clinical decision-making found DeepSeek-R1 achieving 96.7% accuracy on TCM knowledge evaluation and 17.31 out of 20 on clinical case analysis, substantially outperforming GPT-4.5 (52.5%), Claude 3.7 Sonnet (66.3%), and other general-purpose models, while also demonstrating that human–AI collaboration improved decision quality by 16.1% and reduced analysis time by 66.1% [73]. An independent evaluation of LLM adherence to TCM clinical practice guidelines further reported that DeepSeek-R1 and DeepSeek-V3 achieved a median accuracy of 5.00/5.00 in both English and Chinese, surpassing GPT-4o (4.00–4.30) [74]. Beyond these benchmarks, newer-generation models—including Baichuan M3, which excels on general medical benchmarks, the GLM-5 series with improved reasoning capabilities, and Qwen3 with strong few-shot learning stability—have emerged, though systematic TCM-specific benchmarks for these models remain limited at the time of this writing. Collectively, these findings corroborate the core observation from the initial stroke-related study: the gap between knowledge recall and clinical reasoning persists across model generations. The TCM community should therefore interpret LLM capability comparisons as temporally bounded snapshots within a rapidly evolving landscape.

### Chain-of-thought is a reasoning interface, not proof of reasoning quality

The apparent suitability of large language models for traditional Chinese medicine stems largely from their capacity to articulate multi-step reasoning in natural language. Techniques such as chain-of-thought prompting, multi-step reasoning supervision, and staged consultation pipelines align naturally with the diagnostic logic of TCM [23, 24, 26, 32, 33, 48, 52, 53]. Recent TCM-oriented systems explicitly leverage this alignment: HuatuoGPT-o1 was designed to enhance complex medical reasoning through verifier-guided optimization [53], while JingFang emphasizes expert-level syndrome differentiation and introduces a multi-agent reasoning framework [75].

Recent methodological discussions in both TCM and the broader healthcare LLM literature clarify that chain-of-thought generation should not be construed as direct evidence of genuine reasoning [48–55]. A model can produce a plausible multi-step explanation without that chain faithfully reflecting the actual decision process or being grounded in reliable domain knowledge. This underscores the importance of benchmarks like TCMEval-SDT, which explicitly evaluates the syndrome differentiation thought process rather than deeming endpoint correctness sufficient [23]. Consequently, chain-of-thought should be viewed as a reasoning interface, not as proof of successful reasoning.

### Retrieval grounding, knowledge graphs, and GraphRAG as explanation infrastructure

Recent research on TCM LLMs increasingly treats retrieval augmentation, knowledge graphs, and GraphRAG as core explanatory infrastructures, not optional add-ons, because free-form generation is highly prone to hallucination [26, 44, 48–55]. This approach is particularly relevant for TCM, where reasoning often requires explicit linkages among symptoms, syndromes, pathogenesis, classical formula knowledge, and herb compatibility. A clear contemporary example is OpenTCM, which integrates a domain-specific TCM knowledge graph with GraphRAG to enable knowledge retrieval and diagnostic question answering without model fine-tuning [44]. Methodologically, the system's value lies not only in its performance but also in its explicit effort to transform LLM reasoning into an evidence-linked, semantically anchored process. This direction aligns with broader knowledge-centric work in TCM, where the quality of explanation depends heavily on the semantic integrity of the underlying knowledge graph [26, 57–63].

### Multi-turn inquiry and interactive reasoning are becoming part of explainability

A distinctive feature of TCM consultation is its reliance on iterative inquiry rather than static, one-shot prediction. This renders the interactive behavior of an LLM a matter of explainability in itself. When a model can proactively ask relevant follow-up questions, refine its syndrome hypotheses, and expose its uncertainty during a consultation, part of its reasoning becomes inspectable directly through the dialogue. Recent TCM-specific research has begun to address this interactive dimension. For instance, Zhongjing emphasized real-world multi-turn dialogue and expert feedback to improve medical capabilities [52]. More recent work, such as DoPI, proposed a doctor-like proactive interrogation framework for TCM [76]. These systems are significant because they demonstrate that explanation in TCM need not be confined to static feature attribution or final-answer rationalization. Explanation can also emerge through interactive reasoning, where the model reveals uncertainty, clarifies missing information, and makes its inquiry logic visible over time.

### Benchmarking is moving from answer quality toward safety, faithfulness, and clinical utility

A major indicator of a field’s maturation is the increasing alignment of benchmark design with the genuine explanatory requirements of TCM. TCMEval-SDT assesses the thought processes involved in syndrome differentiation [23]. TCMEval-PA evaluates prescription auditing, a task directly linked to medication safety and rational herb use [24]. More recent benchmark proposals further broaden this scope by integrating assessments of syndrome differentiation and treatment with safety and ethical considerations [24, 32, 77]. This progression is crucial, as it shifts evaluation from merely judging endpoint correctness to scrutinizing the validity of reasoning and its relevance to safety.

This shift is necessary because relying solely on answer quality is an insufficient criterion for TCM explainability. A system might generate a plausible syndrome label yet fail to provide a faithful justification, or it could produce a fluent prescription rationale while overlooking critical contraindications. Similarly, it may retrieve factually correct domain information yet still violate fundamental syndrome-treatment coherence. Consequently, high-quality evaluation must determine whether a model's reasoning is clinically applicable, evidence-based, safety-conscious, and semantically consistent with TCM theory [23, 24, 26, 32, 33, 48–55, 77].

### From fluent language to clinically faithful reasoning

Recent research indicates that LLMs represent both the most promising and most perilous approach to TCM-AI explainability. They enable novel applications in natural-language reasoning, patient interaction, consultation support, and knowledge synthesis, yet simultaneously heighten the risk of systems that seem interpretable while being epistemically shallow or clinically unreliable [48–55]. Consequently, the most productive path forward lies not in unrestricted generation, but in reasoning that is grounded, benchmarked, interactive, and semantically constrained. Under this more rigorous standard, LLMs do not replace XAI. Instead, they constitute the context in which the meaning of XAI must evolve from mere transparency toward clinically faithful reasoning (Table 3).

**Table 3:** Representative large language models and evaluation dimensions in Traditional Chinese Medicine

Model	Primary orientation	Explanation mechanism	Evaluation setting/benchmark	Main strength	Main limitation
HuatuoGPT-o1	Complex medical reasoning	CoT-style reasoning externalization, verifier-guided reasoning	Medical reasoning tasks and structured problem-solving settings	Strong structured reasoning and long-chain logic	Faithfulness of generated reasoning remains to be fully validated
Zhongjing	Multi-turn TCM consultation	Interactive inquiry and dialogue-based reasoning	Real-world multi-turn dialogue and expert feedback	Better proactive questioning and information gathering	Limited standardization of explanation evaluation
Qibo	TCM knowledge and domain adaptation	Domain-adapted generation and benchmarked knowledge recall	Qibo benchmark and TCM-specific NLP tasks	Strong domain knowledge and terminology coverage	Reasoning depth may lag behind factual competence
BianCang	Syndrome differentiation and diagnosis	Domain alignment and structured clinical output	Hospital records, pharmacopoeia-oriented tasks, syndrome-related evaluation	Strong diagnostic and syndrome-mapping capability	Explanation quality still depends on external evaluation criteria
Tianyi	General TCM assistant	Benchmark-guided reasoning and broad task coverage	TCMEval and real-world TCM-oriented evaluations	Broad task coverage and strong domain adaptability	Needs stronger validation of clinical faithfulness
OpenTCM	Knowledge-grounded LLM reasoning	GraphRAG, knowledge retrieval, evidence-linked generation	Retrieval and diagnostic QA in TCM knowledge settings	Strong grounding, traceability, and reduced hallucination	Dependent on knowledge graph completeness and domain curation

This table summarizes representative TCM-oriented or TCM-relevant large language models in terms of primary orientation, explanation mechanism, evaluation setting, major strengths, and principal limitations. It emphasizes that LLMs in TCM differ not only in scale or training strategy, but also in the form of explainability they enable.

## Clinical modalities as translation scenarios

### From application catalogues to translation scenarios

Reviews of explainable AI in Traditional Chinese Medicine typically categorize applications into discrete technical domains, such as tongue diagnosis, pulse diagnosis, syndrome differentiation, prescription recommendation, and multimodal fusion. While descriptively useful, this classification fails to capture the core epistemic challenge examined here. Within the semantic translation framework detailed in section "A semantic translation framework for TCM-XAI"—which links data space, computational feature space, and TCM concept space—each application area constitutes a distinct translation scenario [18–21, 26, 30, 32, 33]. Recent reviews of machine learning in TCM diagnosis, multimodal clinical support, and intelligent prescription generation all converge on a common problem: an AI system's success depends not only on its prediction accuracy but also on whether its outputs can be translated into syndrome-consistent, treatment-relevant, and clinically auditable meanings [1–5, 18–21].

This reframing is critical because each modality imposes a unique explanatory burden. Tongue diagnosis primarily involves spatial-semantic translation, connecting image regions to clinically meaningful tongue signs [37–39, 45–47]. Pulse diagnosis centers on tactile-to-computational translation, requiring the conversion of embodied physician touch into machine-readable waveform features that preserve diagnostic meaning [40, 41]. Syndrome differentiation presents a challenge of inferential-semantic translation, as heterogeneous evidence must be organized into pathomechanistic judgment [22–24, 26, 32–36]. For prescription recommendation, the issue shifts to therapeutic and relational translation, where symptoms and syndromes must be linked to treatment principles and herb compatibility via interpretable pathways [56–63]. Finally, multimodal systems require explanations that address cross-modal translation, clarifying how different data streams are weighted, reconciled, and integrated into a final decision [18, 33, 42, 43, 47].

### Tongue diagnosis: from pixels to clinically meaningful signs

Tongue diagnosis represents one of the most established and visible applications of AI in TCM, largely because tongue images are readily digitized, archived, and reviewed. The core challenge for interpretability, however, extends beyond mere image classification or region localization. It involves translating low-level visual patterns—such as color, coating, fissures, tooth marks, shape, localized edge redness, or sublingual features—into clinically meaningful tongue signs and, ultimately, into interpretations relevant to syndromes [18–21, 37–39, 45–47]. Recent formal literature clarifies this progression. Reviews on modernizing tongue diagnosis with AI note a shift from basic image recognition toward explainable, clinically useful feature interpretation [20, 37]. The construction of tongue feature datasets and real-time detection methods addresses a foundational requirement for explainable diagnosis: the standardized annotation of tongue features, without which saliency or classification results lack consistent interpretability [38]. More recent deep-learning systems for automated tongue analysis explicitly connect stable image acquisition, color correction, segmentation, and feature-specific analysis to the objectification and robustness of diagnostic interpretation [39]. These advances are significant not only for improving technical performance but for strengthening the logical chain from image capture to clinically actionable tongue signs.

### Pulse diagnosis: from tactile expertise to waveform semantics

Pulse diagnosis presents a particularly difficult interpretability challenge, as it originates in embodied sensory expertise rather than directly observable images. The central issue is therefore not merely digitization, but whether computationally extracted pulse features can be translated into clinically credible pulse qualities and subsequently into syndrome-relevant reasoning [40, 41]. Recent formal studies demonstrate meaningful progress. For instance, integrating traditional pulse diagnosis with machine learning via intelligent devices has begun to objectify pulse assessment for identifying pregnancy and coronary heart disease [40]. The TPC-GCN model employs multi-domain feature extraction and graph convolution to classify pulse patterns, showing how signal-domain and graph-structured representations can enhance pattern recognition [41]. Nevertheless, pulse diagnosis remains a prime example of why TCM requires XAI as epistemic mediation rather than simple transparency. A model might accurately distinguish waveform classes yet still fail to clarify what those differences signify in terms of practitioner-understood qualities such as wiry, slippery, deep, or thready pulses.

### Syndrome differentiation: translating heterogeneous evidence into pathomechanistic judgment

Syndrome differentiation exemplifies TCM as an interpretive medical system, and thus clearly demonstrates why application-level explainability must be approached as a reasoning problem rather than a classification task. This process requires organizing symptoms, signs, tongue features, pulse findings, medical history, and contextual information into a coherent pathomechanistic judgment to guide treatment [22–24, 26, 32–36]. Recent research increasingly addresses this complexity. The TCM-SD benchmark formalized syndrome differentiation as a language-model probing task using real-world clinical records, while TCMEval-SDT advanced further by explicitly evaluating the reasoning process behind the differentiation, not just the final labels [22, 23]. In applied work, machine learning has been employed to distinguish cold and hot syndromes by integrating TCM and modern clinical variables, illustrating how structured prediction can aid differentiation when explanation relies on symptom combinations rather than isolated markers [34]. Similarly, explainable deep learning has predicted heat syndrome subtypes while revealing the variables driving the model's decisions [36]. Collectively, these studies frame syndrome differentiation as an inferential-semantic translation scenario.

### Prescription recommendation: translating therapeutic logic rather than ranking herbs

Prescription recommendation is often computationally framed as a ranking or generation task, yet in Traditional Chinese Medicine (TCM) it is more precisely a problem of translating therapeutic logic. A clinically meaningful system must therefore predict herbs or formulas associated with symptoms while also preserving the interpretable relationships among symptoms, syndromes, treatment principles, herb functions, and formula structure [56–63].

Recent formal publications substantiate this perspective. For instance, PresRecST explicitly integrates syndrome differentiation and treatment planning into its pipeline, rendering the intermediate clinical reasoning inspectable prior to final prescription generation [56]. The PRDAGE model employs data augmentation and multi-graph embedding to capture symptom–herb relations more comprehensively [62]. Subsequent hierarchical and contrastive models further underscore that recommendation quality hinges on maintaining these structured therapeutic relationships [63]. Collectively, along with graph-based herb recommendation studies [57–61], this work demonstrates that prescription recommendation cannot be sufficiently explained by mere saliency or global importance scores. The necessary explanation must articulate why a particular therapeutic path is chosen, how herb combinations correspond to syndrome and treatment principles, and whether the recommendation is justifiable within the established logic of formula construction.

### Multimodal integration: restoring the four examinations

If any application captures the full epistemic ambition of TCM-AI, it is multimodal integration. TCM diagnosis fundamentally synthesizes multiple information streams, as no single modality adequately represents its clinical reasoning. The core challenge for multimodal AI is thus not merely data fusion, but ensuring the fusion process itself is interpretable [18, 33, 42, 43, 47]. Recent formal studies increasingly address this goal. Reviews on AI in TCM position multimodal fusion as central to next-generation diagnostic and treatment support [18–21]. For instance, multimodal syndrome classification in pediatric allergic rhinitis demonstrates that combining tongue images with clinical features outperforms unimodal models [42]. Earlier multimodal deep architectures for TCM diagnosis remain significant for their explicit attempt to mimic the multimodal perceptual process of TCM practitioners [43]. Collectively, these studies indicate that multimodal integration is the closest computational approximation to the TCM principle of four examinations synthesis, while simultaneously creating the greatest demand for interpretability.

### Clinical applications as real-world tests of epistemic translation

**Fig. 4:**
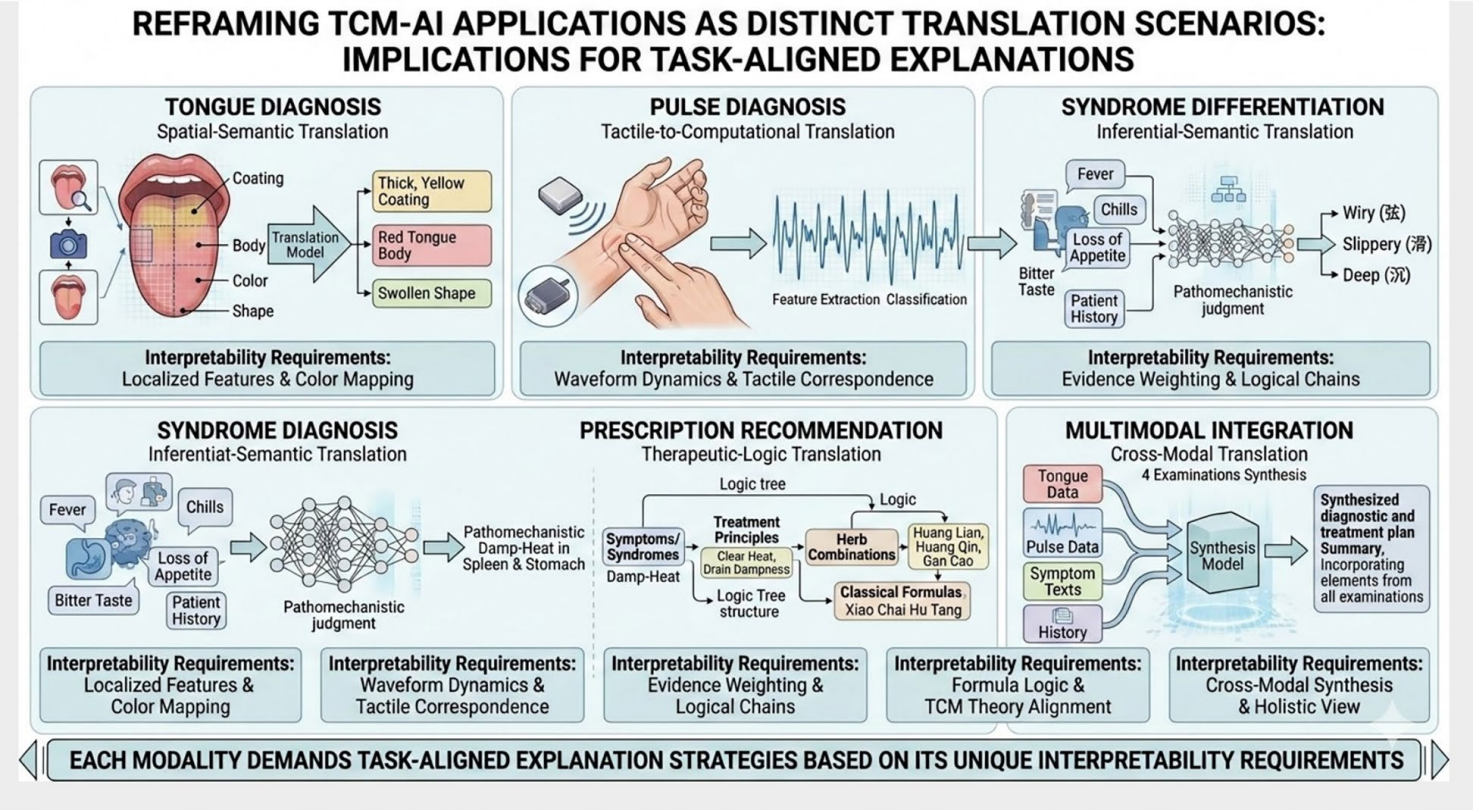
Clinical modalities as epistemic translation scenarios in Traditional Chinese Medicine

In tongue diagnosis, pulse diagnosis, syndrome differentiation, prescription recommendation, and multimodal fusion, the central challenge lies not merely in AI's performance but in how its outputs translate into clinically meaningful TCM reasoning. Consequently, each application serves as a practical test for the semantic translation framework proposed here. Tongue diagnosis examines whether spatial attention maps can be interpreted as clinically relevant tongue signs. Pulse diagnosis evaluates whether extracted waveform features convey meaningful tactile qualities and pathomechanistic insights. Syndrome differentiation assesses whether heterogeneous evidence can be synthesized into a coherent judgment of disease mechanism. Prescription recommendation probes whether a model's therapeutic logic yields an interpretable rationale for herb selection and formula construction. Multimodal integration determines whether information from the four examinations can be fused through an explainable process rather than an opaque latent shortcut. Thus, clinical application constitutes the empirical arena where the success of epistemic translation is ultimately revealed, not simply the endpoint of TCM-XAI development. These modality-specific epistemic translation scenarios are summarized in Fig. 4.

This figure reconceptualizes major TCM-AI applications as distinct translation scenarios instead of isolated technical tasks. Tongue diagnosis constitutes a spatial-semantic translation from image regions to clinically meaningful tongue signs. Pulse diagnosis represents a tactile-to-computational translation from embodied pulse perception into waveform features and pulse categories. Syndrome differentiation is an inferential-semantic translation from heterogeneous evidence to a judgment of pathomechanism. Prescription recommendation is framed as a therapeutic-logic translation that links symptoms, syndromes, treatment principles, and herb combinations. Multimodal integration is depicted as a cross-modal translation problem that approximates the synthesis of the four examinations. The figure emphasizes that each modality imposes distinct interpretability requirements, necessitating task-aligned explanation strategies.

## Challenges beyond accuracy: validation, privacy, fairness, and cultural integrity

### Accuracy is not enough

A recurring limitation in current TCM-AI research is the tendency to treat predictive performance as the dominant indicator of success. As the preceding chapters demonstrate, however, high accuracy alone does not guarantee a system's clinical trustworthiness, theoretical coherence, or cultural legitimacy. The challenge is particularly acute in TCM, where a system is expected not only to predict but to participate in a reasoning tradition structured by syndrome differentiation, treatment principles, and interpretive synthesis [18–24, 26, 32, 33, 48–55]. Recent formal reviews of AI in TCM consistently observe that performance improvements are often easier to demonstrate than explanation faithfulness, multimodal robustness, or semantic consistency with TCM theory [18–21]. Broader work on healthcare AI similarly emphasizes that trustworthy deployment depends on transparency, validation, fairness, and contextual alignment, not merely on accuracy [10, 11, 13, 51, 64–71, 78–90]. Consequently, the central challenges of TCM-XAI can be organized according to the four explanation-quality criteria introduced in section "What counts as a good explanation in TCM?": faithfulness, clinical relevance, theoretical coherence, and cultural integrity.

### Data quality, annotation instability, and the limits of faithfulness

Faithful explanation depends on faithful data and labels, a requirement that is unusually difficult to meet in TCM because its core tasks often rely on heterogeneous, weakly standardized, or expert-dependent clinical data. Variability in tongue imaging conditions, pulse devices, terminology usage, and institutional labeling practices can distort both model learning and post hoc explanations [14, 15, 18–21, 37–43]. More fundamentally, syndrome differentiation itself frequently exhibits inter-rater variability, meaning the ground truth used to train or evaluate a model may encode disagreement rather than a stable medical structure [14, 15]. The expansion of TCM benchmarks and large datasets represents an important advance, yet these resources also reveal the difficulty of creating data that are simultaneously large-scale, clinically rich, and epistemically stable [22–24]. Recent analyses of TCM standardization efforts indicate that international and cross-institutional harmonization remains incomplete, despite a growing number of formal standards [64]. This issue is critical because explainability systems require not only model transparency but also input consistency; if categories such as red tongue, slippery pulse, or qi deficiency are defined variably across datasets or sites, even a technically accurate explanation may be clinically ambiguous.

### Privacy-preserving explainability and the tension between transparency and decentralization

The second major challenge concerns the relationship between explainability and privacy. TCM clinical data are typically fragmented across hospitals, clinics, educational institutions, and region-specific practice networks, where sharing is constrained by privacy regulations, governance limitations, and institutional silos. Consequently, federated learning has become an attractive approach in healthcare and is now widely recognized as a major architectural pathway for privacy-preserving AI [65–67].

Nevertheless, integrating federated learning with explainability introduces a new tension. While TCM-AI requires interpretable reasoning to gain clinical trust, richer explanations may increase the risk of information leakage, expose site-specific biases, or introduce instability across decentralized clients [65–67]. This problem is particularly acute in TCM because explanations often depend on subtle local context, including variations in patient populations, practitioner traditions, device standardization, regional language use, and syndrome-labeling practices. A federated TCM-XAI system must therefore address not only distributed learning but also distributed semantic alignment.

### Fairness, demographic imbalance, and the problem of biased explanation

A third challenge is fairness. As in other medical AI domains, TCM-AI datasets can overrepresent specific demographics, institutions, imaging conditions, or cultural-linguistic groups. Models trained on such imbalanced data may then deliver diagnoses or explanations of differing quality across population subgroups. Broader healthcare research consistently highlights how bias in data, labeling, and deployment can lead to inequitable outcomes, noting that explainability alone does not rectify these biases [68].

This issue is especially pertinent for TCM image analysis. Models for tongue or facial diagnosis can be sensitive to skin tone, lighting, camera specifications, age distribution, or regional phenotypic variation. Similarly, language-based models may inherit biases from dominant corpora, exam-oriented datasets, or highly formalized textbook language that fails to capture authentic patient expression. In such contexts, the explanations themselves can become biased: a model might consistently emphasize different features or assign varying syndrome salience across demographic groups, not due to medically valid distinctions, but because of skewed training distributions.

### Cultural misalignment, theory erosion, and the risk to epistemic integrity

The fourth challenge concerns cultural and epistemic integrity. Traditional Chinese Medicine (TCM) constitutes not merely a clinical system but a historically situated and conceptually distinct medical tradition. As AI systems grow more reliant on language and retrieval, they increasingly mediate the representation, translation, and operationalization of TCM concepts. This mediation risks allowing ostensibly helpful AI to gradually flatten, distort, or oversimplify those concepts to fit computational categories [5, 48–55, 64–71].

Recent formal analyses of cultural bias and alignment in large language models (LLMs) are highly pertinent. Large-scale studies demonstrate that LLMs exhibit measurable cultural biases and can substantially diverge from target cultural value patterns unless specific alignment strategies are implemented [69, 70]. In healthcare, recent reviews similarly underscore that stakeholder alignment, domain grounding, and pluralistic value representation are becoming central to the safe adoption of LLMs [51, 71]. The implications for TCM are serious. Should a language model trained primarily on non-TCM or culturally distant corpora interpret TCM metaphors, pathogenesis terms, or syndrome logic through an alien semantic frame, its explanations may be linguistically polished yet conceptually misleading.

### Toward an evaluation framework beyond accuracy

Taken together, these challenges indicate that evaluating TCM-XAI systems requires moving beyond conventional predictive metrics. As argued in section "What counts as a good explanation in TCM?", a trustworthy system should instead be assessed along four dimensions—faithfulness, clinical relevance, theoretical coherence, and cultural integrity. This expanded framework aligns with recent work in healthcare AI, explainable federated learning, fairness auditing, and cultural alignment [13, 51, 65–71, 78–90]. For TCM specifically, it clarifies why many current systems remain inadequate despite technical sophistication: they may be accurate without being faithful, transparent without being clinically relevant, or explainable without being theoretically coherent.

## Future research agenda: building a trustworthy TCM-AI ecosystem

### From methodological expansion to epistemic consolidation

Future research in explainable artificial intelligence for Traditional Chinese Medicine should move beyond simply generating new models, datasets, or modalities. A more critical objective is epistemic consolidation: creating systems that unify computational performance, clinically meaningful explanations, and theory-consistent reasoning within a coherent framework for evaluation and deployment [18–21, 48–55, 64–71, 78–90]. Contemporary reviews of healthcare XAI increasingly argue that progress depends less on proliferating explanation techniques than on constructing clinically useful, auditable explanation ecosystems that satisfy regulatory and human-factors standards [78–91]. For TCM, this shift implies organizing research around five priorities: causal and counterfactual reasoning, neuro-symbolic and knowledge-grounded architectures, clinician-centered and human-in-the-loop evaluation, standardized multimodal benchmarks and reporting protocols, and regulatory frameworks for explanation quality.

### Causal and counterfactual TCM-AI

A primary challenge is the shift from modeling correlations to reasoning causally and counterfactually. Current TCM-AI research often depends on statistical associations, linking symptoms to syndromes, correlating tongue features with disease labels, recommending herbs from historical patterns, and generating plausible syndrome logic from textual regularities in large language models. Although such approaches can be useful, they cannot adequately address the questions clinicians typically pose: What is the dominant pathogenesis? Which sign reflects the root cause and which is a branch manifestation? How would the treatment strategy change if a key symptom were absent? These are, in essence, counterfactual questions. Recent literature in healthcare increasingly advocates for explainable AI to adopt causally informed reasoning instead of remaining purely correlational [81, 82]. This shift is especially critical for TCM because syndrome differentiation inherently seeks to uncover disease mechanisms rather than merely summarize surface manifestations. Consequently, future TCM-AI systems should progressively integrate causal discovery, counterfactual evaluation, and intervention-aware modeling.

### Neuro-symbolic and knowledge-grounded architectures

A second priority is the development of neuro-symbolic and knowledge-grounded architectures. As this review has argued, a central problem in TCM-AI is the frequent misalignment between purely latent representations and the explicit conceptual structure of TCM. Recent literature in healthcare suggests neuro-symbolic AI offers a route toward more trustworthy and auditable systems by integrating the pattern-learning capabilities of neural models with the explicit reasoning of symbolic representations [83–85]. This approach is particularly well-suited to TCM, a domain already rich in symbolic constructs: syndrome categories, treatment principles, herb functions, formula structures, pathogenesis relations, and classical texts can all provide explicit knowledge constraints. Existing work on knowledge graphs, GraphRAG, and graph-based herb recommendation has demonstrated the utility of such explicit relational structures [26, 44, 57–63]. The necessary progression is from using knowledge as an auxiliary support module to making knowledge-grounded reasoning a core design principle for these systems.

### Clinician-centered and human-in-the-loop evaluation

A third priority involves clinician-centered evaluation, which incorporates human-in-the-loop workflows. Recent medical AI literature increasingly demonstrates that explanation quality cannot be judged solely by technical plausibility, but must also be assessed according to how clinicians use, question, and calibrate their trust in these systems [11, 13, 78–90]. This consideration is especially pertinent in TCM, where an explanation’s value is often determined by its ability to support syndrome reasoning, therapeutic justification, and context-sensitive decision making. Formal recent work on human-in-the-loop AI in healthcare further substantiates this perspective [86, 87]. In TCM, this distinction is critical because practitioners act not merely as end users but also as interpreters of theory, context, and individual patient presentation. Consequently, future TCM-XAI research should integrate prospective clinician studies, workflow-based evaluations, trust calibration analyses, and assessments of cognitive utility, moving beyond a reliance on offline benchmark results alone.

### Standardized multimodal benchmarks and reporting protocols

A fourth priority is the establishment of standardized multimodal benchmarks and reporting standards. As demonstrated in Chapters 5 and 6, the field is progressing toward more comprehensive benchmarks, incorporating tasks such as syndrome-differentiation reasoning, prescription auditing, and multimodal clinical datasets [22–24, 26, 32, 33, 37–43]. These initiatives, however, remain disconnected. No single, widely adopted benchmark framework yet exists to jointly assess multimodal performance, explanation faithfulness, theory alignment, and clinical utility within TCM. This gap reflects a broader challenge in healthcare AI, where recent analyses of reporting standards reveal continued substantial variation in transparency, reporting completeness, and evaluation design across studies [88, 89]. Consequently, future benchmark development for TCM must encompass not only data and task definitions but also gold-standard explanation annotations, modality-specific criteria for explanations, subgroup performance analyses, and explicit reporting of all domain-alignment assumptions.

### Regulatory and governance frameworks for explanation quality

A fifth priority involves developing regulatory and governance frameworks that specifically address explanation quality. The regulatory landscape for medical AI is evolving rapidly, offering concrete precedents that TCM-specific frameworks can build upon. China's National Medical Products Administration (NMPA) has classified medical large language models as high-end medical devices and is developing technical review guidelines that explicitly address algorithmic explainability [12]. The 2026 Expert Consensus on AI Application and Governance in Medical Institutions, endorsed by over 40 leading Chinese medical institutions, establishes explainability as a non-negotiable admission requirement, stipulating that algorithms deemed 'unexplainable' are subject to a one-vote veto and shall not be deployed in clinical settings [92]. The same consensus mandates that for high-risk AI systems—including diagnostic assistance—patients have the right to receive visualization-based explanations translated into plain language, and institutions may not cite 'system calculation' as a defense when a diagnosis is challenged. Internationally, the EU AI Act's risk-categorization framework offers a model for calibrating explainability requirements proportionally: TCM diagnostic AI systems affecting clinical decisions would appropriately be classified as high-risk, warranting mandatory explanation standards, audit trails, and human oversight mechanisms. The November 2025 Implementation Opinions on Promoting and Regulating AI Plus Healthcare, jointly issued by five Chinese ministries including the National Administration of Traditional Chinese Medicine, explicitly identify TCM as one of eight priority directions for AI application, providing a policy mandate for TCM-specific regulatory guidance [93]. Building on these precedents, future TCM governance efforts should address four specific questions: (1) what form of explanation is required for different TCM tasks, with minimum standards differentiated by risk level; (2) what evidence validates explanation quality, including requirements for subgroup performance reporting by age, sex, and disease subtype, consistent with NMPA's existing impact-factor analysis requirements; (3) how explanation failures—such as model flip-flopping, position sensitivity, or culturally inappropriate reasoning—should be audited and remediated; and (4) how cultural or conceptual distortions of TCM knowledge can be prospectively identified and mitigated through pre-deployment review by TCM domain experts.

### Implementation and deployment challenges

A sixth priority involves addressing the practical barriers to deploying XAI systems in real TCM clinical environments. First, computational requirements pose a significant obstacle: LLM-based TCM systems demand substantial GPU resources for inference, while tongue and pulse analysis models may require edge-computing optimization for real-time use in outpatient settings [4, 19, 48–55]. Second, interoperability between AI systems and existing TCM clinical workflows remains underdeveloped. TCM electronic health records lack standardized data formats for storing syndrome differentiation reasoning, pulse-waveform data, or tongue-image annotations, and no widely adopted extension of health-data interchange standards (e.g., HL7 FHIR) yet exists for TCM-specific concepts [64]. Third, clinical workflow integration requires that XAI outputs be presented in formats compatible with real-time decision support rather than as offline retrospective analyses; this in turn demands careful interface design, minimal latency, and clinician training to ensure that explanations augment rather than interrupt diagnostic reasoning [11, 13, 86, 87]. Fourth, cross-institutional deployment faces challenges of annotation variation, device heterogeneity, and differing practice traditions across hospitals and regions, problems that federated learning approaches have begun to address but have not yet resolved [65–67]. Each of these barriers must be systematically addressed if conceptual advances in TCM-XAI are to translate into clinically deployed tools.

### Toward an ecosystem rather than isolated models

Taken together, these priorities indicate that the future of TCM-XAI depends on constructing an integrated ecosystem rather than on isolated model improvements. A trustworthy TCM-AI ecosystem would integrate explanation-ready multimodal datasets, knowledge-grounded and potentially neuro-symbolic architectures, comprehensive benchmark suites for reasoning, safety, and clinical utility, human-in-the-loop evaluation protocols, and governance standards for acceptable explanation quality. This ecosystem perspective is essential, as the central challenge identified in this review—bridging both algorithmic and theoretical opacity—cannot be resolved by any single model or explanation technique. It demands coordinated advances across data, architecture, evaluation, and regulation.

## Conclusion

The integration of artificial intelligence with Traditional Chinese Medicine is frequently framed as a project of technological modernization, yet this review contends that such a characterization is inadequate. The core challenge lies not in applying more powerful algorithms to larger TCM datasets, but in reconciling two fundamentally distinct knowledge systems: one driven by statistical learning and computational abstraction, and the other grounded in holistic, relational, and theory-based medical reasoning [1–5, 7–11, 18–21]. Consequently, AI–TCM integration should be understood not merely as a technical problem, but as an epistemological one.

Building on this premise, the review advances two central arguments. First, it proposes a dual-layer opacity framework (section "The dual-layer opacity problem") to elucidate why TCM presents a uniquely difficult challenge for AI, where algorithmic opacity and theoretical opacity intersect to create a compounded barrier to validation, accountability, and clinical trust. Second, the review argues that explainable artificial intelligence must be reframed for TCM as a process of semantic and epistemic translation, rather than as a narrow transparency tool.

Second, the review argues that explainable artificial intelligence must be reframed for TCM as a process of semantic and epistemic translation, rather than as a narrow transparency tool. Within the TCM context, an explanation is meaningful only when it can mediate between the three representational spaces detailed in section "A semantic translation framework for TCM-XAI", thereby rendering machine-derived outputs clinically interpretable and theoretically coherent [18–24, 26, 32, 33, 45–47, 56–63]. From this perspective, XAI is not a technical add-on appended after prediction; it constitutes the essential translational mechanism that makes AI–TCM integration possible.

The methodological and application-focused sections of this review further demonstrate that no single XAI technique suffices for all TCM tasks. Feature-attribution methods like SHAP and LIME are useful for structured clinical prediction but often remain semantically shallow [28, 29, 34–36]. Visual explanation methods such as Grad-CAM aid in tongue and facial diagnosis yet do not, by themselves, resolve the problem of pathomechanistic meaning [37–39, 45–47]. Process-structured architectures and benchmarked LLM reasoning systems align more closely with syndrome differentiation logic, but they still confront challenges of faithfulness, hallucination, and theory consistency [22–24, 26, 32, 33, 48–55]. Knowledge-guided and graph-based methods appear especially promising because they externalize relational structure in ways that are naturally compatible with TCM reasoning [26, 44, 56–63]. Across all these approaches, a consistent lesson emerges: the success of TCM-XAI depends less on generic transparency than on method–task alignment and translation quality.

This review also contends that the future of TCM-XAI must be evaluated beyond predictive accuracy. The four criteria set out in section "What counts as a good explanation in TCM?"—faithfulness, clinical relevance, theoretical coherence, and cultural integrity—are particularly critical [13, 51, 64–71, 78–90]. A system may be accurate without being faithful, explainable without being clinically useful, transparent without being theory-consistent, or culturally legible without being epistemically robust. In TCM, these distinctions are not peripheral; they determine whether AI will function as a trustworthy collaborator, a fragile automation layer, or a source of epistemic distortion.

Accordingly, the next phase of the field should prioritize causal and counterfactual reasoning, neuro-symbolic and knowledge-grounded architectures, clinician-centered evaluation, explanation-ready multimodal benchmarks, and governance frameworks that define what constitutes an adequate explanation in TCM contexts [81–90]. The goal is not to replace practitioners or reduce TCM to an optimized prediction pipeline, but to build systems capable of supporting human–AI collaborative reasoning in a manner that preserves the interpretive core of TCM while enabling rigorous computational augmentation.

In summary, TCM does not merely require more powerful AI; it requires explainable, theory-aligned, and clinically faithful AI. Only under these conditions can AI transition from being a high-performing but opaque instrument to becoming a legitimate participant in the evolving knowledge system of Traditional Chinese Medicine.

We acknowledge that this review is primarily a conceptual and synthetic contribution, and as such does not include prospective empirical validation of the proposed semantic translation framework in clinical settings. While the framework is grounded in a thorough review of the current literature—including studies demonstrating that explanation quality affects clinician trust [11, 13], that structured intermediate reasoning improves interpretability in prescription recommendation [56], and that knowledge-grounded architectures enhance explanation faithfulness [26, 44, 57–63]—its measurable impact on clinical outcomes, practitioner trust, and diagnostic accuracy remains to be tested. Prospective validation studies are needed to determine whether explanations structured according to the four proposed criteria (faithfulness, clinical relevance, theoretical coherence, and cultural integrity) improve clinician acceptance and decision quality relative to existing explainability approaches. We specifically recommend: (1) controlled experiments comparing clinician diagnostic accuracy and trust calibration with versus without the semantic translation framework; (2) survey studies assessing whether explanations aligned with the four criteria receive higher ratings of usefulness and interpretability from TCM practitioners; and (3) prospective observational studies tracking clinical outcomes in AI-assisted TCM diagnosis across institutions adopting different explainability protocols. Such empirical work represents a necessary next step in translating the conceptual architecture proposed here into clinically validated tools.

## Supplementary Information

Supplementary material 1.

## Data Availability

No datasets were generated or analysed during the current study.

## Abbreviations

AI: Artificial Intelligence
AUC: Area Under the Curve
CNN: Convolutional Neural Network
CoT: Chain-of-Thought
EU: European Union
FHIR: Fast Healthcare Interoperability Resources
GCN: Graph Convolutional Network
GNN: Graph Neural Network
GPU: Graphics Processing Unit
Grad-CAM: Gradient-weighted Class Activation Mapping
GraphRAG: Graph Retrieval-Augmented Generation
HL7: Health Level Seven
KG: Knowledge Graph
LIME: Local Interpretable Model-Agnostic Explanations
LLM: Large Language Model
ML: Machine Learning
NLP: Natural Language Processing
NMPA: National Medical Products Administration
QA: Question Answering
SHAP: SHapley Additive exPlanations
TCM: Traditional Chinese Medicine
XAI: Explainable Artificial Intelligence
XGrad-CAM: Axiom-based Gradient-weighted Class Activation Mapping
