# Supplementary material for: Beyond transparency: why Traditional Chinese Medicine (TCM) need explainable artificial intelligence (XAI)
Source: Chin Med. 2026 Jul 14;21:187. doi: 10.1186/s13020-026-01455-4 (PMC13366969; doi:10.1186/s13020-026-01455-4)
Supplement: Supplementary file 1 — Supplementary material 1. [file 13020_2026_1455_MOESM1_ESM.docx]

**Supplementary Table 1. Empirical performance overview of reviewed TCM-XAI systems.**

The systems discussed in Sections 4 and 6 span diverse tasks and modalities with correspondingly varied performance metrics. In structured clinical prediction, feature-attribution approaches using SHAP have achieved AUC values exceeding 0.85 in TCM efficacy modeling for Long COVID and acute ischemic stroke syndrome classification [33,34]. For tongue diagnosis, deep-learning models incorporating Grad-CAM visualization have reported classification accuracies of 85–95% on specific tongue feature detection tasks, though these metrics are often dataset-dependent and not directly comparable across studies [35–37,47–49]. In prescription recommendation, process-structured models such as PresRecST have demonstrated F1 scores of 0.65–0.78 on real-world prescription prediction, with the crucial advantage of inspectable intermediate reasoning steps [38]. Knowledge-graph-based herb recommendation systems report precision ranging from 0.70 to 0.85 depending on graph density and ontology completeness [39–45]. For LLM-based TCM systems, comprehensive benchmark results are presented in Section 5.2 and Table 3. Across all modalities, a consistent finding emerges: performance gains from architectural complexity do not automatically translate into gains in explanation faithfulness or clinical interpretability, underscoring the central thesis of this review.

| **System / Approach** | **Task / Modality** | **Method** | **Reported Performance** | **Key Limitations** | **Refs** |
| --- | --- | --- | --- | --- | --- |
| SHAP-based structured clinical models | Long COVID efficacy; acute ischemic stroke syndrome classification | Tabular ML + SHAP feature attribution | AUC > 0.85 | Feature importance ≠ TCM-syndrome reasoning; tabular only | [33,34] |
| Grad-CAM tongue diagnosis CNNs | Tongue image classification (color, coating, shape) | Deep CNN + Grad-CAM saliency | Accuracy 85–95% (dataset-dependent) | Saliency rarely maps to clinical tongue concepts; not cross-comparable | [35–37,47–49] |
| PresRecST and process-structured prescription models | Prescription / herb recommendation | Process-structured neural model with intermediate reasoning steps | F1 0.65–0.78 | Reasoning steps still not fully aligned with classical formulae logic | [38] |
| Knowledge-graph-based herb recommendation | Herb / formula retrieval and recommendation | TCM knowledge graph + GNN / path reasoning | Precision 0.70–0.85 | Performance hinges on graph density and ontology completeness | [39–45] |
| LLM-based TCM systems (DeepSeek-R1, GPT-4o, Qibo, BianCang, Tianyi, etc.) | Knowledge QA, syndrome differentiation, clinical case analysis | Domain-adapted LLMs; CoT; GraphRAG | See Section 5.2 and Table 3 (e.g., DeepSeek-R1 96.7% knowledge accuracy; positional inconsistency 0.787→0.470) | Positional sensitivity; knowledge recall ≠ clinical reasoning; rapidly evolving | [28,60,61,29,89–91] |
